# Yeast infections in sterile body fluids: species distribution, antifungal susceptibility, and cross-cutting risk factors for fungemia, sepsis, and mortality (2019–2023)

**DOI:** 10.3389/fmicb.2026.1773098

**Published:** 2026-04-22

**Authors:** Jiaxing Liu, Mei Han, Fan Yang, Shuo Gao, Wanqing Zhou, Han Shen, Xiaoli Cao, Yan Zhang

**Affiliations:** 1 Department of Clinical Laboratory, Nanjing Drum Tower Hospital, Affiliated Hospital of Medical School, Nanjing University, Nanjing, Jiangsu, China; 2 Department of Clinical Laboratory, Jinling Hospital, Affiliated Hospital of Medical School, Nanjing University, Nanjing, Jiangsu, China

**Keywords:** acute kidney injury, antifungal susceptibility, *Candida*, catheter-related infection, echinocandins, fungemia, sepsis, surgical source control

## Abstract

**Background:**

Yeast infections from sterile body fluids are increasingly encountered in tertiary care, yet contemporary links between species ecology, antifungal susceptibility, and patient outcomes remain underdefined.

**Methods:**

We conducted a retrospective cohort study of 231 consecutive sterile-site yeast isolates at Nanjing Drum Tower Hospital (2019–2023). Species were identified by CHROMagar and MALDI-TOF MS; antifungal MICs were determined using YeastOne and interpreted by CLSI M27-Ed3. Clinical data were abstracted from electronic records. Variables with *p* < 0.10 in univariate testing entered multivariable logistic regression to identify independent predictors of fungemia, sepsis, and in-hospital mortality.

**Results:**

The four major Candida species showed distinct susceptibility profiles, with *C. albicans* broadly susceptible, *C. tropicalis* exhibiting notable azole resistance, *Nakaseomyces glabratus* (*C. glabrata*) displaying high azole MICs but good echinocandin activity, and *C. parapsilosis* maintaining low azole MICs but intrinsically higher echinocandin MICs. Clinically, bloodstream involvement was frequent. Fungemia was independently associated with blood transfusion and hemodialysis catheterization, whereas surgery was protective (OR 0.320, 95% CI 0.171–0.599). Sepsis occurred in 23.8% and was independently associated with ICU admission (OR 7.119, 95% CI 2.811–18.026); surgical treatment again showed a protective association (OR 0.426, 95% CI 0.190–0.954). Overall mortality was 26.8%; acute kidney injury (AKI) independently predicted death (OR 3.354, 95% CI 1.563–7.198).

**Conclusions:**

In this five-year cohort, *Candida*—led by *C. albicans* with a rising *non-albicans* share—dominated sterile-site infections. Echinocandins and amphotericin B retained broad activity, whereas azole activity was reduced in *C. tropicalis/N. glabratus*. Across outcomes, transfusion and hemodialysis catheters increased fungemia risk, ICU admission increased sepsis risk, AKI drove mortality, and early surgical source control was consistently protective. These findings support prompt device removal, restrictive transfusion, proactive renal management, and echinocandin-first strategies in high-risk patients.

## 1 Introduction

Invasive fungal disease (IFD) has emerged as a major cause of morbidity and mortality among hospitalized and immunocompromised patients worldwide (Denning, 2024). Over the past decade, the incidence of invasive yeast infections, particularly those caused by *Candida* species, has increased substantially due to the widespread use of broad-spectrum antibiotics, immunosuppressive therapy, invasive medical procedures, and the growing population of critically ill patients (GBD 2023 Cancer Collaborators, 2025; Chamorro et al., 2025). Although *Candida albicans* remains the predominant pathogen, infections caused by non-*albicans Candida* (NAC) species such as *N. glabratus, C. tropicalis*, and *C. parapsilosis* have risen significantly, often exhibiting decreased susceptibility to commonly used azole antifungals (Siopi et al., 2020). These trends pose serious challenges to clinical management and infection control.

Sterile body fluids—including blood, cerebrospinal fluid, pleural effusion, ascitic fluid, pericardial effusion, synovial fluid, and bile—represent normally sterile compartments of the human body. The isolation of yeast from these specimens is clinically significant and typically indicates invasive infection rather than contamination (Pappas et al., 2016). However, due to nonspecific clinical manifestations and the slow turnaround time of culture-based diagnostics, timely diagnosis and appropriate antifungal therapy remain difficult, leading to delayed treatment and poorer patient outcomes.

Antifungal resistance among *Candida* species, particularly azole resistance, is a growing global concern (Li Y. et al., 2025). Continuous surveillance of species distribution and antifungal susceptibility is therefore critical to guide empirical therapy and monitor emerging resistance trends. Moreover, the identification of clinical risk factors associated with sepsis, fungemia, and mortality in IFD patients can provide valuable insights for early risk stratification and optimized management.

This study retrospectively analyzed yeast isolates recovered from sterile body fluids at Nanjing Drum Tower Hospital between 2019 and 2023. We aimed to (1) characterize the species distribution and antifungal susceptibility patterns of the isolates, and (2) evaluate clinical features and risk factors associated with sepsis, fungemia, and mortality. The findings are expected to contribute to a better understanding of the epidemiology and clinical determinants of invasive yeast infections, supporting improved diagnostic and therapeutic strategies in clinical practice.

## 2 Materials and methods

### 2.1 Yeast isolates

A total of 231 yeast isolates were obtained from sterile body fluid specimens collected at Nanjing Drum Tower Hospital between January 2019 and December 2023. The samples were collected from blood (n=105), peritoneal (ascitic) fluid (n=66), bile (n=14), cerebrospinal fluid (n=5), pleural fluid (n=2), and synovial (joint) fluid (n=1). The numbers of isolates collected each year were 12 in 2019, 29 in 2020, 36 in 2021,37 in 2022, and 117 in 2023. An increasing trend in the number of isolates was observed over the study period, particularly with a marked rise in 2023. All isolates were initially cultured and presumptively identified using CHROMagar Candida plates (CHROMagar, France), and species-level identification was confirmed using Matrix-Assisted Laser Desorption/Ionization Time-of-Flight Mass Spectrometry (MALDI-TOF MS) (bioMérieux, France), which was consistently available and routinely used throughout the entire study period, following the manufacturer's instructions. Each isolate represented a single, unique patient; when multiple isolates were obtained from the same patient, only the first isolate was included in the analysis. Since only isolates from sterile body fluids were included, the total number of cases was relatively limited despite the tertiary hospital setting.

### 2.2 Antifungal susceptibility testing

Antifungal susceptibility testing was performed using Sensititre YeastOne antifungal susceptibility plates (Thermo Fisher Scientific, USA) according to the manufacturer's instructions (Espinel-Ingroff et al., 1999). The minimum inhibitory concentrations (MICs) endpoints were determined based on the colorimetric change (from red to blue), which allows standardized and objective interpretation and reduces the subjectivity associated with visual growth assessment. MICs were determined for the following antifungal agents: fluconazole, voriconazole, itraconazole, posaconazole, amphotericin B, caspofungin, micafungin, anidulafungin, isavuconazole and flucytosine. Interpretation of susceptibility results followed the Clinical and Laboratory Standards Institute (CLSI) M27-Ed3 guidelines (Clinical and Laboratory Standards Institute, 2008). Quality control was ensured using *C. parapsilosis* ATCC 22019 and *C. krusei* ATCC 6258 as reference strains.

### 2.3 Clinical data collection

Clinical data of patients with yeast isolates from sterile body fluids were retrospectively collected from the electronic medical record system of Nanjing Drum Tower Hospital. The collected variables included demographic information (age, sex), underlying diseases (such as diabetes mellitus, malignancy, chronic kidney disease, liver disease, cardiovascular disease, and immunosuppressive conditions), hospitalization details (ward type, length of stay, intensive care unit [ICU] admission), and clinical outcomes.

The onset of IFD was defined as the date when the first positive sterile body fluid culture yielding a yeast isolate was obtained. Sepsis was diagnosed based on two criteria: (1) the presence of a confirmed or suspected infection, and (2) an increase in the Sequential Organ Failure Assessment (SOFA) score of at least two points from baseline (Singer et al., 2016). Invasive procedures during hospitalization were defined as therapeutic or diagnostic interventions, including vascular or urinary catheter insertion, mechanical ventilation, bronchoscopy, colonoscopy, surgical operations, and blood transfusions (Centers for Disease Control and Prevention (CDC), 2023; Wyss et al., 2023; ASGE Quality Assurance in Endoscopy Committee, 2018; Karstensen et al., 2022). Procedures performed within 10 days prior to the onset of infection were considered potential risk factors for transient fungemia (Merck Manual Professional, 2024). Information on antifungal exposure and immunosuppressive therapy within the 30 days prior to infection was also collected. This included chemotherapy, corticosteroid therapy (≥10 mg/day of prednisone or equivalent for >10 days), and systemic antifungal therapy (any antifungal agent administered for >48 hours) (Marty et al., 2022). Antifungal treatment was considered appropriate if the yeast isolate was susceptible to at least one antifungal drug that had been administered.

### 2.4 Risk factor analysis

To evaluate potential risk factors associated with adverse clinical outcomes, patients were stratified into three groups: sepsis vs. non-sepsis, based on the diagnostic criteria mentioned earlier; fungemia vs. non-fungemia, depending on the presence of yeast isolates in blood cultures; and death vs. survival, according to patient outcomes at discharge. Comparative analyses across these groups were performed to identify clinical, microbiological, and therapeutic factors potentially associated with sepsis, fungemia, or mortality in patients with yeast infections.

### 2.5 Statistical analysis

All data were processed and analyzed using SPSS software version 27.0 (IBM Corp., Armonk, NY, USA), with additional data management performed using Microsoft Excel 2017 (Microsoft Corp., USA) and WHONET version 5.6 (World Health Organization, Geneva, Switzerland) for antimicrobial susceptibility data. Continuous variables were presented as median values with interquartile ranges [M (P_2_5, P75)], while categorical variables were expressed as counts and percentages. Univariate analyses were performed using the chi-square test or two-tailed Fisher's exact test, as appropriate, to identify potential risk factors associated with invasive yeast infections. Variables with *p* < 0.10 in univariate analysis were subsequently included in a multivariate logistic regression model to determine independent risk factors. All statistical tests were two-tailed, and a p-value < 0.05 was considered statistically significant.

## 3 Results

### 3.1 Distribution of yeast isolates and sources of sterile body fluid samples (2019–2023)

From 2019 to 2023, a total of 231 cases of fungal infections in sterile body fluids were identified at Nanjing Drum Tower Hospital. The majority of infections were caused by *Candida* species (96.1%, 222/231), with no isolates of *Aspergillus* spp. or *Candida auris* detected during the study period. The remaining 3.9% (9/231) of infections were caused by non-Candida yeasts, including *Cryptococcus neoformans* (n = 5), *Kodamaea ohmeri* (n = 3), and *Rhodotorula mucilaginosa* (n = 1).

**Figure 1:**
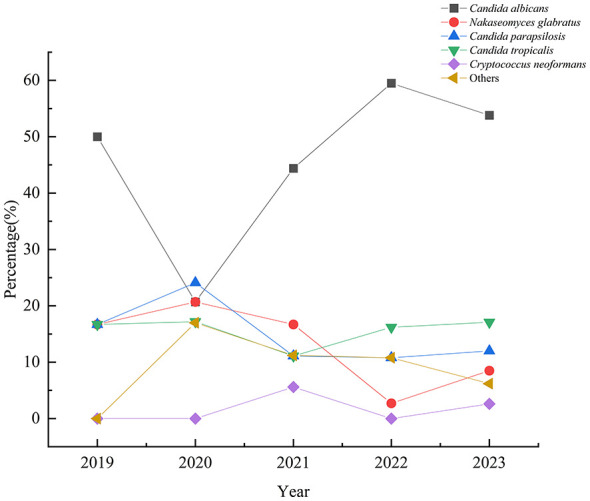
Distribution of 231 yeasts isolated from aseptic body fluid samples from 2019 to 2023. The figure illustrates the prevalence of different yeast species over the five-year period, with percentages indicating the proportion of each species within the total yeast isolates.

**Figure 2:**
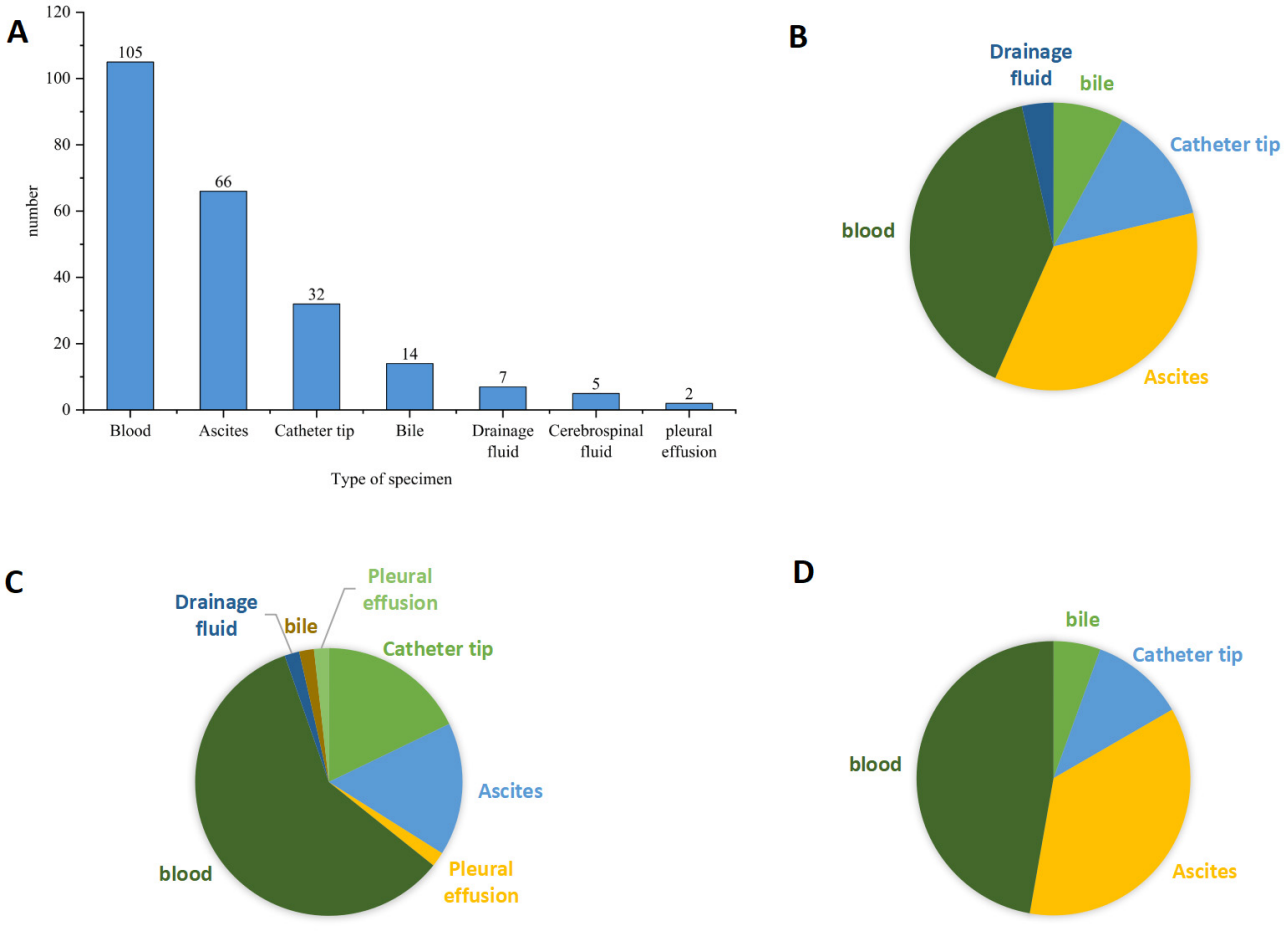
Distribution of clinical specimens for different *Candida* species. **(A)** Distribution of specimen types for all 231 clinical yeast strains, including blood ascites, catheter tip, bile, drainage fluid, cerebrospinal fluid, and pleural effusion. **(B)** Distribution of clinical specimen types for *Candida albicans* isolates. **(C)** Distribution of clinical specimen types for *Candida parapsilasis* isolates. **(D)** Distribution of clinical specimen types for *Candida tropicalis* isolates.

Among the *Candida* isolates, *C. albicans* was the most frequently identified species (48.9%). From 2019 to 2023, the distribution of yeast species among clinical isolates showed temporal variability. *C. albicans* remained the dominant species, accounting for about half of isolates overall, with a decline in 2020, a peak in 2022, and a slight decrease in 2023. NAC species varied more markedly: *N. glabratus* and *C. parapsilosis* increased in 2020 and then declined or stabilized, while *C. tropicalis* showed a gradual increase from 2021 onward and became more prevalent in later years. *Cryptococcus neoformans* remained uncommon, peaking in 2021 with a decline in 2022 before rebounding in 2023. Other rare yeast species, including *K. ohmeri* and *R. mucilaginosa*, contributed only sporadic cases throughout the study period. Overall, the findings indicate a dynamic shift in species composition with persistent predominance of *C. albicans* (Figure 1). Further, *Candida* isolates were most frequently recovered from blood samples, followed by ascites and catheter tips, while bile, drainage fluid, cerebrospinal fluid, and pleural effusion contributed relatively few isolates (Figure 2A). *C. albicans* was predominantly isolated from blood and ascites, indicating its major role in bloodstream and intra-abdominal infections, with fewer isolates obtained from catheter tips and other specimen types (Figure 2B). *C. tropicalis* also showed a predominance in blood samples but exhibited a comparatively higher proportion in catheter tips and ascites, suggesting a broader distribution in invasive and device-associated infections (Figure 2C). *C. parapsilosis* was mainly recovered from blood, with notable representation in ascites and catheter tip specimens, consistent with its known association with intravascular devices (Figure 2D).

### 3.2 Antifungal susceptibility patterns of major *Candida* species (2019–2023)

The MIC distributions of ten antifungal agents against the four *Candida* species are summarized as follows (Tables 1, 2). Antifungal susceptibilities were interpreted according to CLSI clinical breakpoints or epidemiological cutoff values (ECVs) where available, and isolates were classified as susceptible (S), intermediate or susceptible-dose dependent (I/SDD), and resistant (R), or as wild-type (WT) and non–wild-type (NWT), accordingly. The corresponding proportions (%) for each category are shown in Tables 1, 2.

**Table 1:** Antifungal susceptibility of four fungal species across various drug concentrations (0.015–256μg/ml).

Fungal species	0.015	0.03	0.06	0.12	0.25	0.5	1	2	4	8	16	32	64	128	256	MIC50	MIC90	S/WT (%)	I/SDD (%)	R/NWT (%)
Amphotericin B																				
*C. tropicalis* (*n* = 37)					2	16	19									1	1	37 (100%)		
*C. albicans* (*n* = 113)					1	75	37									0.5	1	113 (100%)		
*N. glabratus* (*n* = 25)					2	17	6									0.5	1	25 (100%)		
*C. parapsilosis* (*n* = 31)					6	16	9									0.5	1	31 (100%)		
Fluconazole																				
*C. tropicalis* (*n* = 37)							9	13	8					3	4	2	128	22 (59.5%)	8 (21.6%)	7 (18.9%)
*C. albicans* (*n* = 113)				2	31	46	20	4	2	4		1	1	2		0.5	2	103 (91.2%)	2 (1.7%)	8 (7.1%)
*N. glabratus* (*n* = 25)							1	6	4	6	7	1				8	16		25 (100%)	
*C. parapsilosis* (*n* = 31)					1	14	11	5								1	2	31 (100%)		
Flucytosine																				
*C. tropicalis* (*n* = 37)			21	7	2				7							0.06	4	NA	NA	NA
*C. albicans* (*n* = 113)			54	38	2	4	3		11		1					0.12	4	NA	NA	NA
*N. glabratus* (*n* = 25)			16					1	6		1	1				0.06	4	NA	NA	NA
*C. parapsilosis* (*n* = 31)			14	7	1	9										0.12	0.5	TR-L	TR-L	TR-L
Itraconazole																				
*C. tropicalis* (*n* = 37)				7	12	11	5	1	1							0.25	1	30 (81.1%)		7 (18.9%)
*C. albicans* (*n* = 113)	3	14	47	25	11	8	1	2	1	1						0.06	0.5	NA	NA	NA
*N. glabratus* (*n* = 25)			1	3	4	13	4									0.5	1	25 (100%)		
*C. parapsilosis* (*n* = 31)		8	6	15	2											0.12	0.12	31 (100%)		

NA, no corresponding MIC or ECV data according to CLSI cutoffs (not available); TR-L, ECV cannot be defined for this species-antifungal agent combination, because minimal inhibitory concentration distribution is truncated on the low end of the recommended testing range; S, susceptible; I, intermediate; R, resistant; SDD, susceptible-dose dependent; WT, wild type; NWT, non-wild type.

**Table 2:** Antifungal susceptibility of four fungal species across various drug concentrations (0.008- 1 μg/ml).

Fungal species	0.008	0.015	0.03	0.06	0.12	0.25	0.5	1	2	4	8	MIC_50_	MIC_90_	S/WT (%)	I/SDD (%)	R/NWT (%)
Voriconazole																
*C. tropicalis* (*n* = 37)				6	13	10	1				7	0.12	8	19 (51.3%)	11 (29.7%)	7 (19.0%)
*C. albicans* (*n* = 113)	57	26	6	12	1	5	3		1		2	0.008	0.12	102 (90.3%)	8 (7.1%)	3 (2.6%)
*N. glabratus* (*n* = 25)			1	5	3	10	5	1				0.25	0.5	19 (76.0%)		6 (24.0%)
*C. parapsilosis* (*n* = 31)	5	12	3	10	1							0.015	0.06	31 (100%)		
Posaconazole																
*C. tropicalis* (*n* = 37)			7	1	2	10	9	7	1			0.25	1	10 (27.0%)		27 (73.0%)
*C. albicans* (*n* = 113)		24	60	19	1	1	4	3			1	0.03	0.06	103 (91.2%)		10 (8.8%)
*N. glabratus* (*n* = 25)			8			1	3	11	2			1	1	23 (92.0%)		2 (8.0%)
*C. parapsilosis* (*n* = 31)		2	21	6	1	1						0.03	0.06	31 (100%)		
Isavuconazole																
*C. tropicalis* (*n* = 37)	24	9		1	1			1			1	0.008	0.06	NA	NA	NA
*C. albicans* (*n* = 113)	84	17	1	2	2	2	1	1	2		1	0.008	0.03	NA	NA	NA
*N. glabratus* (*n* = 25)	18	4				1			2			0.008	0.25	NA	NA	NA
*C. parapsilosis* (*n* = 31)	25	2		1	1	1	1					0.008	0.06	NA	NA	NA
Anidulafungin																
*C. tropicalis* (*n* = 37)		2	6	11	18							0.06	0.12	37 (100%)		
*C. albicans* (*n* = 113)		13	34	46	19				1			0.06	0.12	112 (99.1%)		1 (0.9%)
*N. glabratus* (*n* = 25)		2	10	12				1				0.06	0.06	24 (96.0%)		1 (4.0%)
*C. parapsilosis* (*n* = 31)				9		2	9	10				0.5	1	31 (100%)		
Caspofungin																
*C. tropicalis* (*n* = 37)			5	13	15	4						0.12	0.25	37 (100%)		
*C. albicans* (*n* = 113)			39	36	35	2	1					0.06	0.12	112 (99.1%)	1 (0.9%)	
*N. glabratus* (*n* = 25)			3	10	11	1						0.06	0.12	24 (96.0%)	1 (4.0%)	
*C. parapsilosis* (*n* = 31)				9		5	15	2				0.5	0.5	31 (100%)		
Micafungin																
*C. tropicalis* (*n* = 37)		7	21	9								0.03	0.06	37 (100%)		
*C. albicans* (*n* = 113)	23	81	6	1		1			1			0.015	0.015	112 (99.1%)		1 (0.9%)
*N. glabratus* (*n* = 25)		22	2				1					0.015	0.03	24 (96.0%)		1 (4.0%)
*C. parapsilosis* (*n* = 31)		9				2	6	9	5			0.5	2	31 (100%)		

NA, no corresponding MIC or ECV data according to CLSI cutoffs (not available); S, susceptible; I, intermediate; R, resistant; SDD, susceptible-dose dependent; WT, wild type; NWT, non-wild type.

According to CLSI criteria, the vast majority of *C. albicans* isolates remained susceptible to agents with defined breakpoints or ECVs, including fluconazole, voriconazole, posaconazole, echinocandins, and amphotericin B. MICs for isavuconazole were uniformly low (mostly 0.008 mg/L), and itraconazole MICs mainly ranged between 0.03 and 0.25 mg/L; however, clinical breakpoints are not established for *C. albicans* for these two azoles. Fluconazole MICs predominantly ranged from 0.25 to 1 mg/L, correlating with high susceptibility (91.2% S) and a low resistance rate (7.1%). Amphotericin B MICs were around 0.25–1 mg/L. Echinocandins, particularly micafungin, demonstrated high activity, with over 99% of isolates classified as S or WT. Flucytosine MICs were also determined, but clinical breakpoints are not defined for this species.

In contrast, *C. tropicalis* demonstrated a broader MIC distribution for several azoles. Fluconazole resistance was observed in 18.9% of isolates, with an additional 21.6% classified as SDD, indicating a trend toward reduced susceptibility. Voriconazole and posaconazole also showed increased proportions of NWT isolates. MICs for isavuconazole varied, but interpretive criteria are not available for *C. tropicalis*. Amphotericin B MICs were generally between 0.25–1 mg/L. Flucytosine MICs were determined, though clinical breakpoints are not available. Echinocandins showed varied susceptibility patterns.

*N. glabratus* exhibited the highest rates of reduced susceptibility to fluconazole, voriconazole, and posaconazole among the four species. MICs for these azoles were substantially higher compared to other species, with fluconazole reaching up to 32 mg/L. Isavuconazole MICs were also elevated, but no clinical breakpoints exist for *N. glabratus*. However, echinocandins remained largely effective, with >95% of isolates classified as WT, although occasional NWT isolates were detected, particularly for caspofungin and micafungin. Amphotericin B showed similar MIC values as for *C. albicans*. Flucytosine MICs were determined; however, clinical breakpoints are not established for *N. glabratus*.

For *C. parapsilosis*, MICs to fluconazole, voriconazole, and posaconazole were generally low, except for a broader itraconazole range. Isavuconazole MICs were also low, but interpretive criteria are lacking. Amphotericin B MICs were mainly between 0.25–1 mg/L. While this species displayed intrinsically higher MICs to echinocandins compared to the others, all isolates remained within the WT category according to CLSI ECVs. For the *C. parapsilosis*-flucytosine combination, ECVs cannot be defined because the MIC distribution exhibits a testing range-low (TR-L) pattern. This occurs when the MIC distribution is truncated at the low end of the recommended testing range, preventing reliable statistical demarcation of the wild-type population.

The analysis of MIC50 and MIC90 revealed marked species-specific differences. *C. albicans* remained highly susceptible to most antifungal agents with defined breakpoints, while *C. tropicalis* exhibited elevated MIC90 values to fluconazole and voriconazole, indicating pronounced reduced susceptibility to these agents. *N. glabratus* showed reduced susceptibility to key azoles but retained good susceptibility to echinocandins. Notably, *C. parapsilosis* exhibited higher MICs to echinocandins, though it remained susceptible to most azoles for which breakpoints exist.

Overall, echinocandins demonstrated the most consistent *in vitro* activity across species based on available ECVs, while azole susceptibility varied substantially by species, particularly among *C. tropicalis* and *N. glabratus*, underscoring the importance of species-level identification and the cautious interpretation of susceptibility results in the context of available CLSI guidelines.

### 3.3 Clinical characterization

Among the 231 patients with yeast isolates from sterile body fluids, most were male (69.3%, 160/231), with an age range of 14–95 years and a median age of 66 years. Nearly half had a history of surgery (46.3%, 107/231) and were primarily admitted to surgical wards, followed by the ICU, internal medicine departments, and emergency services. All patients had at least one underlying comorbidity, and many underwent surgical procedures (63.2%) or received blood transfusions (45.0%). Indwelling catheters were required in 96.3% of cases, most commonly urinary (87.2%) and vascular catheters (86.3%), with invasive interventions documented in 89.4% (219/231) before the onset of invasive fungal disease (IFD). Nearly all patients (96.6%) received antimicrobial therapy within 1 month before IFD onset, while only 18 had prior antifungal exposure. Additionally, 24 patients (10.1%) underwent chemotherapy, and 20 (9.6%) received corticosteroids. For antifungal prophylaxis, fluconazole was most frequently prescribed (23 cases), followed by caspofungin (20) and voriconazole (17).

Fungemia patients were generally older and more likely to be male (63.8%), though these differences were not statistically significant (*P* > 0.05). A greater proportion were admitted to the ICU (59.0%), reflecting more severe clinical conditions. Underlying diseases, including malignancy (34.3%), acute kidney injury (29.5%), and pulmonary infection (45.7%), were more common in the fungemia group compared with non-fungemia patients, while other chronic comorbidities such as hypertension and diabetes showed no significant differences.

Among 231 patients with yeast isolates from sterile body fluids, 55 (23.8%) developed sepsis. Sepsis patients were generally older, with over half aged >65 years (55.4%), and were more likely to be admitted to the ICU (72.7%). They frequently had underlying malignancy (29.1%), acute kidney injury (38.2%), and pulmonary infection (81.8%), which were significantly more common than in the non-sepsis group (*P* < 0.05). Most sepsis cases occurred in critically ill or surgical patients, who often underwent invasive procedures, such as vascular catheterization (81.8%), urinary catheterization (89.1%), mechanical ventilation (54.5%), and surgery (49.1%). Nearly all patients received antibiotic therapy (94.8%) within 1 month prior to infection.

Compared with survivors, non-survivors were slightly older, with 54.8% aged over 65 years, and had a higher rate of ICU admission (40.3% vs. 32.1%), although this difference was not statistically significant (*P* = 0.092). They exhibited a significantly higher prevalence of acute kidney injury (41.9% vs. 14.2%, *P* < 0.001), pulmonary infection (61.3% vs. 35.5%, *P* = 0.002), and chronic renal failure (32.3% vs. 12.4%, *P* = 0.004). Malignancy (38.7% vs. 24.9%) and cardiovascular disease (38.7% vs. 26.6%) were also more common among non-survivors, though the differences were not statistically significant. Invasive procedures such as urinary catheterization (75.8%), mechanical ventilation (53.2%), and surgery (54.8%) were frequent, and blood transfusion (59.7% vs. 40.8%, *P* = 0.023) was significantly associated with mortality. Overall, non-survivors tended to be older, critically ill patients with renal and pulmonary complications, and were more likely to undergo invasive interventions and transfusions, suggesting that organ dysfunction and intensive treatment were closely linked to poor outcomes.

### 3.4 Multivariate analysis of clinical risk factors associated with sepsis, fungemia, and mortality

Univariate analysis identified ICU admission, malignancy, pulmonary infection, biliary tract infection, acute kidney injury, and surgical intervention as factors significantly associated with sepsis. In multivariate logistic regression, ICU admission emerged as a strong independent risk factor for sepsis (OR = 7.119, 95% CI: 2.811–18.026, *P* < 0.001), whereas surgical treatment demonstrated a protective effect (OR = 0.426, 95% CI: 0.190–0.954, *P* = 0.038; Table 3).

**Table 3:** Univariate analysis and multivariate analysis for the risk factors of patients with non-sepsis and sepsis.

Variables	Non-sepsis (*n* = 176)	Sepsis (*n* = 55)	Total (*n* = 231)	Univariate analysis		Multivariate analysis	
				OR (95% CI)	*P*-value	OR (95% CI)	*P*-value
Demographics							
Male gender	117 (66.5%)	30 (54.5%)	147 (63.6%)	0.605 (0.327–1.121)	0.108		
Age ≥ 65 years	94 (53.4%)	32 (58.2%)	126 (54.5%)	1.214 (0.658–2.239)	0.535		
Admission to Intensive care unit (ICU)	80 (45.5%)	48 (87.3%)	128 (55.4%)	8.229 (3.529–19.188)	< 0.001	7.119 (2.811–18.026)	**0.000**
Underlying conditions							
Hypertension	82 (0.18)	18 (32.7%)	100 (43.3%)	0.558 (0.295–1.054)	0.070	0.553 (0.261–1.173)	0.122
Diabetes mellitus	49 (27.8%)	14 (25.5%)	63 (27.3%)	0.885 (0.444–1.765)	0.729		
Autoimmune disease	13 (7.4%)	2 (3.6%)	15 (6.5%)	0.473 (0.103–2.165)	0.502		
Malignancy	79 (44.9%)	16 (29.1%)	95 (41.1%)	0.504 (0.262–0.968)	0.038	0.869 (0.391–1.930)	0.730
Cerebrovascular disease	49 (27.8%)	17 (30.9%)	66 (28.6%)	1.160 (0.599–2.244)	0.660		
Cardiovascular disease	64 (36.4%)	21 (38.2%)	85 (36.8%)	1.081 (0.579–2.019)	0.807		
Presence of chronic renal failure (CRF)	26 (14.8%)	12 (21.8%)	38 (16.5%)	1.610 (0.750–3.454)	0.219		
Acute kidney injury(AKI)	22 (12.5%)	27 (49.1%)	49 (21.2%)	6.750 (3.378–13.486)	< 0.001	3.776 (1.727–8.260)	**0.001**
Pulmonary infection	62 (35.2%)	34 (61.8%)	96 (41.6%)	2.977 (1.592–5.566)	< 0.001	1.801 (0.861–3.766)	0.118
Urinary tract infection	20 (11.4%)	10 (18.2%)	30 (13.0%)	1.733 (0.757–3.969)	0.189		
Biliary tract infection	27 (15.3%)	15 (27.3%)	42 (18.2%)	2.069 (1.006–4.257)	0.045	1.729 (0.715–4.180)	0.224
Invasive procedures during hospital stay							
Vascular catheter	142 (80.7%)	45 (81.8%)	187 (81.0%)	1.077 (0.494–2.352)	0.851		
Urinary catheter	141 (80.1%)	49 (89.1%)	190 (82.3%)	2.027 (0.804–5.112)	0.128		
Mechanical ventilation	95 (54.0%)	34 (61.8%)	129 (55.8%)	1.380 (0.743–2.565)	0.307		
Invasive surgery	118 (67.0%)	27 (49.1%)	145 (62.8%)	0.474 (0.256–0.877)	0.016	0.426 (0.190–0.954)	**0.038**
Blood transfusion	80 (45.5%)	29 (52.7%)	109 (47.2%)	1.338 (0.730–2.456)	0.346		
Treatments within 1 month							
Antibiotic therapy	166 (94.3%)	53 (96.4%)	219 (94.8%)	1.596 (0.339–7.516)	0.804		

The bold values represents the variables that are statistically significant in the multivariate analysis.

For fungemia, univariate analysis revealed significant associations with acute kidney injury (*P* = 0.030), hemodialysis catheter use (*P* = 0.018), surgical procedures (*P* = 0.003), and blood transfusion (*P* = 0.006). Multivariate analysis confirmed that both blood transfusion and hemodialysis catheterization were independent risk factors (both *P* < 0.05), while surgical procedures were independently associated with a reduced risk of fungemia (OR = 0.320, 95% CI: 0.171–0.599, *P* < 0.001; Table 4).

**Table 4:** Univariate analysis and multivariate analysis for the risk factors of patients with non-fungemia and fungemia.

Variables	Non-fungemia (*n* = 126)	Fungemia (*n* = 105)	Total (n = 231)	Univariate analysis		Multivariate analysis	
				OR (95% CI)	*P*-value	OR (95% CI)	*P*-value
Demographics							
Male gender	84 (66.7%)	63 (60.0%)	147 (63.6%)	0.750 (0.438–1.285)	0.294		
Age ≥ 65 years	70 (55.6%)	56 (53.3%)	126 (54.5%)	0.914 (0.544–1.538)	0.736		
Admission to Intensive care unit (ICU)	66 (52.4%)	62 (59.0%)	128 (55.4%)	1.311 (0.777–2.211)	0.310		
Underlying conditions							
Hypertension	60 (0.18)	40 (38.1%)	100 (43.3%)	0.677 (0.400–1.146)	0.146		
Diabetes mellitus	34 (27.0%)	29 (27.6%)	63 (27.3%)	1.033 (0.577–1.846)	0.914		
Autoimmune disease	8 (6.3%)	7 (6.7%)	15 (6.5%)	1.054 (0.369–3.008)	0.922		
Malignancy	59 (46.8%)	36 (34.3%)	95 (41.1%)	0.592 (0.347–1.010)	0.054		
Cerebrovascular disease	30 (23.8%)	36 (34.3%)	66 (28.6%)	1.670 (0.940–2.967)	0.079	1.233 (0.666–2.282)	0.505
Cardiovascular disease	41 (32.5%)	44 (41.9%)	85 (36.8%)	1.495 (0.873–2.560)	0.142		
Presence of chronic renal failure (CRF)	17 (13.5%)	21 (20.0%)	38 (16.5%)	1.603 (0.796–3.228)	0.184		
Acute kidney injury (AKI)	20 (15.9%)	29 (27.6%)	49 (21.2%)	2.022 (1.065–3.841)	0.030	1.415 (0.711–2.817)	0.323
Pulmonary infection	48 (38.1%)	48 (45.7%)	96 (41.6%)	1.368 (0.809–2.316)	0.242		
Urinary tract infection	15 (11.9%)	15 (14.3%)	30 (13.0%)	1.233 (0.572–2.658)	0.592		
Biliary tract infection	25 (19.8%)	17 (16.2%)	42 (18.2%)	0.780 (0.396–1.539)	0.474		
Invasive procedures during hospital stay							
Vascular catheter	95 (75.4%)	92 (87.6%)	187 (81.0%)	2.309 (1.137–4.689)	0.018	2.350 (1.061–5.207)	**0.035**
Urinary catheter	104 (82.5%)	86 (81.9%)	190 (82.3%)	0.957 (0.487–1.884)	0.900		
Mechanical ventilation	67 (53.2%)	62 (59.0%)	129 (55.8%)	1.270 (0.752–2.143)	0.371		
Invasive surgery	90 (71.4%)	55 (52.4%)	145 (62.8%)	0.440 (0.255–0.758)	0.003	0.320 (0.171–0.599)	**0.000**
Blood transfusion	49 (38.9%)	60 (57.1%)	109 (47.2%)	2.095 (1.237–3.549)	0.006	2.237 (1.240–4.034)	**0.007**
Treatments within 1 month							
Antibiotic therapy	119 (94.4%)	100 (95.2%)	219 (94.8%)	1.176 (0.362–3.821)	0.787		

The bold values represents the variables that are statistically significant in the multivariate analysis.

Regarding mortality, univariate analysis identified chronic renal failure (*P* = 0.005), acute kidney injury (*P* = 0.002), pulmonary infection (*P* = 0.038), and blood transfusion (*P* = 0.012) as significant predictors. In the multivariate model, acute kidney injury remained the most significant independent predictor of death (OR = 3.354, 95% CI: 1.563–7.198, *P* = 0.002; Table 5).

**Table 5:** Univariate analysis and multivariate analysis for the risk factors of survival and mortality in IFI patients.

Variables	Survival (*n* = 169)	Mortality (*n* = 62)	Total (*n* = 231)	Univariate analysis		Multivariate analysis	
				OR (95% CI)	*P*-value	OR (95% CI)	*P*-value
Demographics							
Male gender	104 (61.5%)	43 (69.4%)	147 (63.6%)	1.414 (0.759–2.636)	0.274		
Age ≥ 65 years	94 (55.6%)	32 (51.6%)	126 (54.5%)	0.851 (0.475–1.525)	0.588		
Admission to Intensive care unit (ICU)	88 (52.1%)	40 (64.5%)	128 (55.4%)	1.674 (0.917–3.054)	0.092	1.112 (0.539–2.290)	0.774
Underlying conditions							
Hypertension	75 (0.18)	25 (40.3%)	100 (43.3%)	0.847 (0.469–1.530)	0.581		
Diabetes mellitus	49 (29.0%)	14 (22.6%)	63 (27.3%)	0.714 (0.361–1.412)	0.729		
Autoimmune disease	9 (5.3%)	6 (9.7%)	15 (6.5%)	1.905 (0.649–5.591)	0.234		
Malignancy	73 (43.2%)	22 (35.5%)	95 (41.1%)	0.723 (0.396–1.322)	0.291		
Cerebrovascular disease	45 (26.6%)	21 (33.9%)	66 (28.6%)	1.411 (0.754–2.642)	0.280		
Cardiovascular disease	56 (33.1%)	29 (46.8%)	85 (36.8%)	1.773 (0.980–3.208)	0.057	1.112 (0.545–2.267)	0.770
Presence of chronic renal failure (CRF)	21 (12.4%)	17 (27.4%)	38 (16.5%)	2.662 (1.294–5.477)	0.006	3.141 (1.422–6.936)	**0.005**
Acute kidney injury (AKI)	24 (14.2%)	25 (40.3%)	49 (21.2%)	4.082 (2.097–7.949)	< 0.001	3.354 (1.563–7.198)	**0.002**
Pulmonary infection	60 (35.5%)	36 (58.1%)	96 (41.6%)	2.515 (1.388–4.559)	0.002	2.027 (1.041–3.944)	**0.038**
Urinary tract infection	20 (11.8%)	10 (16.1%)	30 (13.0%)	1.433 (0.630–3.260)	0.189		
Biliary tract infection	28 (16.6%)	14 (22.6%)	42 (18.2%)	1.469 (0.715–3.018)	0.294		
Invasive procedures during hospital stay							
Vascular catheter	137 (81.1%)	50 (80.6%)	187 (81.0%)	0.973 (0.465–2.036)	0.943		
Urinary catheter	143 (84.6%)	47 (75.8%)	190 (82.3%)	0.570 (0.278–1.166)	0.120		
Mechanical ventilation	96 (56.8%)	33 (53.2%)	129 (55.8%)	0.865 (0.482–1.552)	0.627		
Invasive surgery	110 (65.1%)	35 (56.5%)	145 (62.8%)	0.695 (0.384–1.258)	0.229		
Blood transfusion	69 (40.8%)	40 (64.5%)	109 (47.2%)	2.635 (1.440–4.820)	0.001	2.292 (1.197–4.388)	**0.012**
Treatments within 1 month							
Antibiotic therapy	161 (95.3%)	58 (93.5%)	219 (94.8%)	0.720 (0.209–2.483)	0.852		

The bold values represents the variables that are statistically significant in the multivariate analysis.

## 4 Discussion

This study provides a comprehensive analysis of yeast isolates obtained from sterile body fluids over a five-year period (2019–2023) at Nanjing Drum Tower Hospital, offering valuable insights into the evolving epidemiology, antifungal susceptibility, and clinical characteristics of IFDs. The clinical importance of these findings lies in the fact that yeast infections in sterile body fluids—such as blood, cerebrospinal fluid, or ascitic fluid—typically indicate true invasive infection rather than colonization or contamination, and are frequently associated with high morbidity and mortality (Denning, 2024). Identifying the dominant *Candida* species, tracking resistance trends, and recognizing key clinical risk factors can facilitate early diagnosis, guide targeted therapy, and improve clinical outcomes for critically ill patients. Since our analysis was restricted to yeast isolates obtained from sterile body fluids, which represent only a subset of invasive fungal infections, the overall number of cases may appear relatively limited despite the tertiary hospital setting.

Notably, the number of yeast isolates increased substantially over the study period, rising from 12 cases in 2019 to 117 cases in 2023, a trend that is likely multifactorial. Population aging may have played an important role, as patients aged ≥65 years accounted for more than half of all cases (54.5%, 126/231); older individuals are more susceptible to invasive fungal infections due to immunosenescence and a higher burden of comorbidities such as chronic obstructive pulmonary disease and diabetes mellitus (Chong et al., 2022). In addition, advances in critical care medicine may have contributed to this increase. The intensive care unit (ICU) represented the second most common department associated with infection (29.4%, 68/231), and improved survival of critically ill patients may prolong hospital stays and increase exposure to nosocomial pathogens, thereby expanding the window for opportunistic fungal infections (Samuelsen et al., 2024). Clinical intervention–related factors are also likely contributors, including the widespread use of broad-spectrum antibiotics, corticosteroids, and other immunosuppressive agents, as well as the increasing application of invasive procedures such as central venous catheterization, parenteral nutrition, and mechanical ventilation, all of which facilitate fungal colonization and subsequent bloodstream or deep-seated infections (Roberts et al., 2025). Finally, the post-2019 COVID-19 pandemic period may have exerted an indirect impact: COVID-19–associated immune dysregulation and residual pulmonary impairment may predispose patients to secondary fungal infections (Bistagnino et al., 2024), while heightened awareness of infection prevention and control and strengthened antimicrobial stewardship policies may have increased microbiological surveillance and clinically indicated culture submissions, thereby improving the detection of invasive yeast infections. Taken together, these findings support a genuine increase in invasive yeast infections rather than merely improved detection.

In agreement with national and international surveillance data, *C. albicans* remained the predominant species in our study (48.9%), though the proportion of NAC species showed a rising trend, especially *C. tropicalis* and *N. glabratus* (Denning, 2024; Żyrek et al., 2021; Byun et al., 2023). Similar epidemiological shifts have been reported in large multicenter studies from China, Europe, and the United States, where the incidence of NAC infections has gradually surpassed *C. albicans* in certain patient populations (Ghojoghi et al., 2022; Abdel-Hamid et al., 2023; Aboagye et al., 2025). This shift is often attributed to prolonged antibiotic use, increasing immunocompromised hosts, and the widespread application of invasive devices—all of which create selective pressure favoring NAC species. Notably, no *Candida auris* isolates were identified during the study period. However, in our ongoing institutional surveillance, the first case of *C. auris* infection was detected in 2024, suggesting its recent emergence in our region.

Recent antifungal surveillance data from China and international programs reveal both shared patterns and notable regional differences in *Candida* susceptibility, and our findings are largely concordant with these reports when interpreted using CLSI criteria. In line with global surveillance data, *C. albicans* in our cohort remained broadly susceptible to azoles, echinocandins, and amphotericin B, with uniformly low MIC distributions and a high fluconazole susceptibility rate (91.2%) (Cornely et al., 2025; Rodríguez Stewart et al., 2024; Ordaya et al., 2023). Notably, MICs for both isavuconazole and itraconazole were low, although formal clinical breakpoints are not yet established for *C. albicans*, limiting definitive clinical interpretation of these results. These results confirm that *C. albicans* continues to be the most predictable species in terms of antifungal susceptibility in invasive infections. In contrast, *C. tropicalis* exhibited a pronounced shift toward reduced azole susceptibility. Nearly one-fifth of isolates were classified as fluconazole-resistant, with an additional proportion categorized as SDD, accompanied by elevated MIC90 values for fluconazole and voriconazole. This pattern closely mirrors recent Chinese multicenter studies reporting increasing azole resistance and the emergence of high-level fluconazole-resistant *C. tropicalis* isolates, whereas resistance rates in Europe and North America remain comparatively low and stable (Tseng et al., 2024; Fan et al., 2023a,b). These findings suggest region-specific selective pressure, potentially driven by antifungal exposure patterns. *N. glabratus* demonstrated the highest degree of reduced azole susceptibility among the four major species, characterized by universally elevated fluconazole MICs and frequent increases in voriconazole and posaconazole MICs. As with *C. albicans*, elevated MICs were also observed for isavuconazole against *N. glabratus*, but the absence of CLSI breakpoints precludes a definitive resistance assessment for this agent. Nevertheless, echinocandins largely retained *in vitro* activity against *N. glabratus* in our study, with the majority of isolates classified as WT, consistent with globally reported trends (Singh-Babak et al., 2012; Lotfali et al., 2021). Occasional NWT isolates to anidulafungin and micafungin underscore the importance of continued resistance surveillance in this species. Caspofungin susceptibility testing is known to be associated with significant interlaboratory variability, and therefore its results should be interpreted with caution. In addition, echinocandin resistance cannot be definitively established without molecular confirmation of FKS mutations. In this context, anidulafungin is generally considered a more reliable phenotypic indicator of echinocandin resistance. Therefore, the observed NWT isolates in our study, particularly for caspofungin and micafungin, should be interpreted as reduced susceptibility rather than confirmed resistance. *C. parapsilosis* showed low resistance rates to azoles and favorable fluconazole susceptibility; however, as expected, it exhibited intrinsically higher echinocandin MICs compared with other *Candida* species. Although all isolates in our cohort remained within CLSI WT ranges, reports from China have described increasing echinocandin MICs and sporadic pan-echinocandin–resistant strains, highlighting a potential emerging concern (Ordaya et al., 2023; Ning et al., 2023; Sang et al., 2025). A specific methodological observation for this species was the consistent TR-L pattern for flucytosine, wherein the MIC distribution was truncated at the low end of the concentration tested. This statistical phenomenon is the recognized reason CLSI has not defined an ECV for the *C. parapsilosis*-flucytosine combination, as it precludes reliable separation of WT and NWT populations. Collectively, these species-specific susceptibility patterns—characterized by stable susceptibility in *C. albicans*, rising azole resistance in *C. tropicalis*, intrinsic azole tolerance in *N. glabratus*, and reduced echinocandin susceptibility in *C. parapsilosis*—underscore the necessity of species-level identification and CLSI-guided antifungal susceptibility testing. Our study also highlights scenarios where current CLSI interpretive criteria are lacking, particularly for isavuconazole and itraconazole against several species, and for flucytosine against *C. parapsilosis* due to the TR-L pattern. These gaps emphasize the need for caution when interpreting MIC data in the absence of established breakpoints or ECVs, and point to areas requiring further clinical-epidemiological correlation to inform future guideline development. Integrating local epidemiological data into empirical and targeted antifungal therapy remains essential to optimize clinical outcomes.

Consistent with prior national and international studies, the majority of patients in our cohort were elderly, with a median age of 66 years, and predominantly male (69.3%) (Gong et al., 2020; Shekhova et al., 2024; Yamin et al., 2021). Age has long been recognized as an important risk factor for invasive fungal infections due to declining immunity and increased comorbidity burden. Similar age distributions have been reported in studies from the United States and Europe, where older adults constitute the majority of invasive candidiasis cases (Rayens et al., 2022; Yazdi et al., 2023). Nearly all patients in our cohort had at least one underlying disease, with malignancy, chronic organ dysfunction, and metabolic disorders being the most frequent—consistent with previous findings that immunosuppression and chronic illness substantially increase the risk of fungal infection (Biyun et al., 2024). Our study revealed an extremely high rate of invasive device use, with 96.3% of patients having indwelling catheters and 89.4% undergoing invasive procedures before infection onset. This aligns with previous studies showing that central venous and urinary catheters are major predisposing factors for IFD (Monzó-Gallo et al., 2023; Thomas-Rüddel et al., 2022; Mora et al., 2024; Medina-Polo et al., 2021). The mechanical disruption of epithelial barriers, coupled with biofilm formation on catheter surfaces, facilitates yeast colonization and subsequent bloodstream invasion (La Bella et al., 2023; Kalinina and Wilson, 2025). The strong association between ICU admission, mechanical ventilation, and IFD in our study further supports the established notion that critical illness and intensive procedures increase fungal infection risk. Nearly all patients (96.6%) had received broad-spectrum antibiotics prior to infection, reflecting the well-recognized link between antibiotic overuse and fungal overgrowth. Similar patterns have been observed in studies from Italy, Japan, and India, where prolonged antibiotic therapy disrupts the normal bacterial flora and promotes fungal proliferation (Saha et al., 2022; Auchtung et al., 2022; Most et al., 2024). Only a small fraction of patients (7.8%) had prior antifungal exposure, suggesting that empirical antifungal prophylaxis remains limited in practice. Among those who received prophylaxis, fluconazole was the most frequently prescribed agent, in line with its common use in high-risk surgical and ICU populations (Cornely et al., 2025). However, considering the increasing prevalence of NAC with reduced azole susceptibility, fluconazole-based prophylaxis should be carefully evaluated, and echinocandins may be preferred in high-risk or azole-resistant settings.

Fungemia, identified in 45.5% of cases, was strongly linked to vascular catheterization and transfusion—findings consistent with international reports that central venous access is the leading cause of candidemia (Sang et al., 2025; Gong et al., 2020; Shekhova et al., 2024); notably, surgical intervention appeared protective, likely because early surgical source control can reduce fungal burden and systemic spread (Wiggins and Reddy, 2022). Sepsis occurred in 23.8% of patients—primarily among older adults and those admitted to ICUs—echoing multicenter data showing that invasive fungal sepsis is prevalent in critically ill surgical populations (Alenazy et al., 2021; Bassetti et al., 2024); within our cohort, acute kidney injury, malignancy, and pulmonary infection were prominent, suggesting a multifactorial interplay between immune dysregulation, organ dysfunction, and nosocomial factors. Finally, the overall mortality rate (26.8%) was comparable to that reported in other tertiary centers (Guo et al., 2024; Li C. et al., 2025); non-survivors tended to be older, with a higher prevalence of renal impairment, pulmonary infection, and transfusion history, aligning with reports from Europe and North America that identify renal failure and respiratory involvement as key predictors of death in IFD patients (Rhodes, 2025; Baden et al., 2024; Paiva and Pereira, 2023).

Consistent with findings from Chinese tertiary hospitals and multicenter surveillance in Europe and the United States (Monzó-Gallo et al., 2023; Thomas-Rüddel et al., 2022; Mora et al., 2024), fungemia was independently associated with central vascular catheterization and blood transfusion—mechanisms likely driven by catheter biofilm persistence and transfusion-related immunomodulation—whereas surgical intervention appeared protective when it enabled early, definitive source control and shortened exposure to invasive devices; sepsis, in turn, was linked to ICU admission and AKI in agreement with domestic IFD reports (e.g., CHIF-NET subanalyses) and international ICU cohorts (e.g., EPIC II), as invasive procedures and broad-spectrum antibiotic exposure disrupt mucosal barriers while renal dysfunction compromises immunity and alters antimicrobial pharmacokinetics (Bai et al., 2021; Li et al., 2023; Pais et al., 2024); mortality in our cohort (26.8%) fell within domestic (~25–40%) and Western (~20–35%) ranges, with AKI the strongest predictor of death—followed by chronic renal failure, pulmonary infection, and transfusion (Rhodes, 2025; Baden et al., 2024; Paiva and Pereira, 2023)—mirroring European and North American data and suggesting that the more pronounced net protective effect of surgery in our setting reflects timelier source control and improved perioperative infection management; overall, evidence across regions converges that renal impairment, invasive devices, and transfusion are cross-cutting risk factors for fungemia, sepsis, and death, whereas timely surgical source control can mitigate these risks and supports strategies emphasizing early catheter removal, restrictive transfusion, and proactive renal optimization in high-risk ICU patients.

This single-center retrospective study has several limitations. Its design may limit generalizability and introduce potential selection and information bias. SOFA scores were assessed only at hospital admission, preventing evaluation of the temporal relationship between SOFA changes and culture positivity. Culture-based case ascertainment may also have led to misclassification of colonization as infection, while fungemia and sepsis were not uniformly adjudicated in all cases. In addition, time-dependent exposures, including catheter management, timing of antifungal therapy, surgical source control, and transfusion, were not modeled, introducing potential immortal-time bias and confounding by indication. The appropriateness of antifungal therapy was assessed solely according to in vitro susceptibility, because detailed treatment data were incomplete. Finally, susceptibility testing was based on YeastOne MICs interpreted using CLSI criteria without therapeutic drug monitoring or molecular resistance testing; notably, *FKS* mutation analysis was not performed, which may limit the definitive interpretation of echinocandin resistance.

## 5 Conclusion

In our study, *Candida* spp. predominated, with *C. albicans* most common (48.9%) and a rising non-albicans burden—especially *C. tropicalis*. Echinocandins and amphotericin B remained highly active overall, whereas azole susceptibility was reduced in *C. tropicalis*/*N. glabratus*, and *C. parapsilosis* showed modestly lower echinocandin activity. Clinically, bloodstream infection was frequent: fungemia was independently associated with blood transfusion and hemodialysis catheters, whereas surgery reduced risk; sepsis was independently associated with ICU admission, with surgery again protective; and AKI emerged as the strongest independent predictor of death. These data highlight the need to limit unnecessary transfusions and invasive devices, prioritize early surgical source control, choose echinocandins or amphotericin B when appropriate, and closely monitor renal function in high-risk patients.

## Data Availability

The raw data supporting the conclusions of this article will be made available by the authors, without undue reservation.
